# Integrative analysis of Anoikis-related genes reveals that FASN is a novel prognostic biomarker and promotes the malignancy of bladder cancer via Wnt/β-catenin pathway

**DOI:** 10.1016/j.heliyon.2024.e34029

**Published:** 2024-07-03

**Authors:** Ruoyu Peng, Xiaohan Ma, Zhiyun Jiang, Yu Duan, Shaogang Lv, Wei Jing

**Affiliations:** a Department of Clinical Laboratory, The First Affiliated Hospital of Zhengzhou University, Key Laboratory of Laboratory Medicine of Henan, Zhengzhou, 450000, China; b Department of Laboratory Medicine, The Third Affiliated Hospital of Zhengzhou University, Zhengzhou Key Laboratory for In Vitro Diagnosis of Hypertensive Disorders of Pregnancy, Zhengzhou, 450000, China

**Keywords:** Bladder cancer, Anoikis-related genes, FASN, Wnt/β-catenin pathway, Biomarker, Machine learning

## Abstract

Bladder cancer (BC) exhibits diversity in clinical outcomes and is characterized by heterogeneity. Anoikis, a form of programmed cell death, plays a crucial role in facilitating tumor invasion and metastasis. This study comprehensively investigated the genetic landscape of BC progression, identifying 300 differentially expressed Anoikis-related genes (DE-ARGs) through in-depth analysis of the GSE13507 datasets. Functional enrichment analysis revealed associations with diverse diseases and biological processes. Employing machine learning algorithms, a logistic regression model based on nine marker genes demonstrated superior accuracy in distinguishing BC from normal samples. Validation in TCGA datasets highlighted the prognostic significance of LRP1, FASN, and SIRT6, suggesting their potential as cancer biomarkers. Particularly, FASN emerged as an independent prognostic indicator, regulating BC cell proliferation and metastasis through the Wnt/β-catenin pathway. The study provides crucial insights into altered genetic landscapes and potential therapeutic strategies for BC, emphasizing the significance of FASN in BC prognosis and progression.

## 1 Introduction

With a tenfold higher risk of occurrence compared to women, bladder cancer (BC) is the tenth most prevalent cancer worldwide and the sixth most prevalent in men [1]. BC has an increasing incidence with age, particularly in the age group of 60 and above. Elderly individuals are more susceptible to the influence of chronic diseases, environmental exposures, and genetic factors [2]. Exposure to certain chemical substances in occupational environments, such as benzene and dimethylamine, is associated with the occurrence of BC [3]. Certain industries, such as dyeing, rubber, and leather manufacturing, may be correlated with an increased risk of BC [4,5]. In addition, smoking is one of the primary risk factors for BC. Harmful substances in tobacco smoke can be transported to the bladder through the bloodstream, increasing the risk of developing BC. Smokers have a significantly higher risk of BC compared to non-smokers [6]. Despite advancements in treatment choices, effectively managing the disease remains challenging, given its elevated rates of recurrence and metastasis, along with its inherent heterogeneity. Consequently, it is imperative to pinpoint relevant biomarkers with predictive value for treatment response and clinical outcomes, aiming to forge tailored therapeutic strategies.

In the process of cancer development, metastasis constitutes a crucial aspect of its malignant progression. Metastasis involves the intra-body migration and proliferation of cancer cells, with the involvement of the Extracellular Matrix (ECM) playing a pivotal role in this process [7,8]. The ECM not only provides structural support to cells but also regulates cell behavior by participating in cell signaling pathways [9]. During the metastatic process, the interaction between cancer cells and the ECM plays a crucial role. However, once tumor cells detach from the ECM, a specific form of programmed cell death known as Anoikis is triggered [10,11]. Anoikis is a programmed cell death that occurs when cells lose their connection with the extracellular matrix, and it is of critical importance in maintaining tissue structure and preventing the migration of cancer cells to other sites. In the mechanisms of tumor invasion and metastasis, the inhibition or evasion of Anoikis plays a significant role [12,13]. Cancer cells demonstrate the ability to evade Anoikis, employing various strategies to maintain their survival and continue the process of metastasis. These strategies include the utilization of growth factors, activating survival signals through growth factor signaling pathways to suppress Anoikis [14,15]. Additionally, the regulation of the pH levels in the cell's surrounding environment is a manifestation of cancer cell adaptability, mitigating the adverse effects of Anoikis [16,17]. Adapting to oxidative stress is also a crucial aspect of cancer cell survival and the metastatic process, achieved by modulating antioxidant systems. Interestingly, recent research suggests that anoikis-related genes (ARGs) may play a significant role in cancer therapy [18,19]. ARGs belong to a category of genes that respond to stress in the extracellular environment and may be closely associated with processes such as maintaining cell survival and preventing apoptosis. In-depth studies of ARGs could provide new insights for the development of novel strategies in cancer treatment. This discovery offers a fresh perspective on cancer treatment research and applications, holding the potential to provide more effective therapeutic approaches for patients in the future.

This study conducted an analysis using the GSE13507 dataset, identifying 300 differentially expressed ARGs (DE-ARGs) that were predominantly enriched in various diseases. Utilizing machine learning algorithms, nine potential biomarkers were determined, and a logistic regression model based on these markers exhibited excellent performance in distinguishing BC from normal samples. Our findings delved into the diagnostic and prognostic value of FASN in BC, demonstrating its independence as a prognostic indicator and constructing a diagnostic model for comprehensive assessment of patient survival prospects. Finally, we explored the potential function of FASN in BC. This comprehensive study provides genetic and functional insights for the diagnosis, treatment, and prognosis of BC, offering new directions for future cancer research and therapy.

## 2 Materials and methods

### 2.1 TCGA-BLCA cohort and GEO cohort

Through the Cancer Genome Atlas (TCGA) data portal (https://gdc-portal.nci.nih.gov/), we successfully obtained Level-three transcriptome RNA sequencing data from patients with BC(including 19 normal samples and 412 tumor samples) and collected relevant clinicopathological features. Additionally, we extracted RNA sequence information from normal and BC samples using the GSE13507 (67 normal samples and 165 tumor samples) and GSE3167 (9 normal samples and 41 tumor samples) datasets, laying the groundwork for further analysis. Moreover, we employed MSigDB (https://www.gsea-msigdb.org/gsea/msigdb) for screening and identified 508 Anoikis-related genes (ARGs). These ARGs may play a crucial role in the development and progression of BC, providing compelling clues for a deeper understanding of its molecular mechanisms.

### 2.2 Differential expression analysis

We conducted a comprehensive analysis of the expression profiles of 508 ARGs obtained from the GSE13507 database, encompassing both normal and BC samples. Utilizing the R platform and employing Student's t-test, we successfully identified differentially expressed ARGs (DE-ARGs) between these two sample sets. Employing a threshold of a p-value less than 0.05, we specifically defined genes showing significant differential expression between the normal and BC groups as DE-ARGs. This stringent screening process ensured our focus on genes that may play crucial roles in Anoikis regulation, allowing for in-depth exploration of their potential functions in the development of BC.

### 2.3 Functional enrichment of DE-ARGs

“clusterProfiler” is an R package designed for bioinformatics and biostatistics analyses, primarily focusing on functional enrichment and pathway analysis [20]. The main purpose of this package is to assist researchers in understanding the biological significance of differentially expressed genes within large-scale gene expression datasets. The “clusterProfiler” package provides a robust toolkit that enables researchers to systematically analyze gene expression data, gaining insights into the functionality of differentially expressed genes and their roles in biological processes. This is crucial for interpreting results from biological experiments, comprehending gene functions, and identifying key pathways associated with diseases [21]. We utilized the “clusterProfiler” package in the R language to conduct in-depth exploration of the functional and pathway associations of the genes through Gene Ontology (GO) and Kyoto Encyclopedia of Genes and Genomes (KEGG) pathways. Comprehensive functional annotations in biological processes, cellular components, and molecular functions were performed using the “clusterProfiler” package. Additionally, we accurately identified the key signaling pathways involved in the DE-ARGs. For the Disease Ontology (DO) enrichment analysis of DE-ARGs, we employed the “clusterProfiler” and “DOSE” packages in R. “DOSE” is a package designed for bioinformatics and biostatistics analysis in the R language. Its primary purpose is to facilitate functional enrichment analysis using DO, aiding researchers in understanding the associations between gene sets and specific diseases. In the analysis, we set the minimum gene set to 5, the maximum gene set to 5000, and established statistical significance criteria with a significance level of P < 0.05 and a false discovery rate (FDR) < 0.1.

### 2.4 Analysis of DEGs between FASN-high and -low expression BC groups

In the analysis of BC groups, we aimed to identify Differentially Expressed Genes (DEGs) based on the expression levels of FASN. The “limma” package was employed for this purpose, allowing us to pinpoint genes with distinct expression patterns in individuals with high and low FASN expression. These identified DEGs constitute valuable candidates for further investigation. To visually represent the results, a heat map was generated. These graphical tools provide a clear and intuitive depiction of the expression differences among the identified DEGs.

### 2.5 Construction of the LASSO model and SVM-RFE feature selection process

Firstly, the LASSO regression method was employed using the “glmnet” package, with the response type set to binomial and an alpha value of 1, to screen genes exhibiting significant differences in the diagnosis of BC. This step distinctly identified potential biomarkers, contributing to precise BC diagnosis. Secondly, the Support Vector Machine Recursive Feature Elimination (SVM-RFE) algorithm was introduced as a supervised machine learning approach. SVM-RFE is a machine learning algorithm used for feature selection. It is a variant of Support Vector Machine (SVM) that focuses on iteratively eliminating less relevant features from a dataset. The goal is to find the subset of features that contributes the most to the predictive performance of the SVM classifier. Through iteratively eliminating irrelevant features, it gradually identified crucial biomarkers contributing to BC diagnosis. SVM-RFE utilized the SVM classifier from the R package at http://github.com/johncolby/SVM-RFE, incorporating k-fold cross-validation to ensure model robustness and generalizability. Throughout the entire selection process, the number of features was halved in each round until fewer than 100 remained, ensuring that the selected biomarkers possessed better interpretability and applicability. Finally, by overlapping the genes obtained from both algorithms, a final set of candidate genes was determined, considered to have higher reliability and importance in the diagnosis of BC. To further validate these candidate genes, researchers turned to the GSE3167 dataset, conducting an examination of the expression levels of these genes. The overarching aim of this study design was to integrate the strengths of LASSO regression and SVM-RFE to enhance the accuracy of BC diagnosis. Through validation on the GSE3167 dataset, the study reinforced the reliability and robustness of the candidate genes across multiple experimental datasets.

### 2.6 Construction and evaluation of the nomogram model

Firstly, we conducted a thorough univariate Cox regression analysis to explore the potential prognostic value of critical diagnostic genes and clinical-pathological features in patient outcomes. This step aimed to identify factors with significant impacts in survival analysis, encompassing gene expression levels and clinical-pathological information. Subsequently, for a comprehensive understanding of the independent contributions of each factor to patient prognosis, we performed a multivariate Cox regression analysis. Through this step, we could further filter out key factors that maintained prognostic significance even when considering other variables, thereby obtaining a more reliable predictive model. Following that, we constructed a nomogram model using the “rms” package and the “survival” package in the R language. This model synthesized multiple factors influencing patient prognosis, including critical diagnostic genes and clinical-pathological features. The establishment of the nomogram aimed to provide an intuitive and effective tool to assist clinicians in evaluating the individualized prognosis of patients. Finally, to comprehensively assess the performance of the nomogram model, we employed metrics such as the concordance index (C-index), ROC curves, calibration curves, and decision curve analysis (DCA).

### 2.7 Cell culture and transfection reagents

We utilized the SV40 immortalized uroepithelium cell line SVHUC-1 and human bladder cell lines T24, SW780, 5637, RT4, and J82. The selection of these cell lines was based on their widespread application and reliability in relevant research areas. All cell lines were sourced from the American Type Culture Collection (ATCC), with suppliers claiming regular testing for mycoplasma contamination to ensure the reliability of experiments and the credibility of data. To investigate the role of FASN in cell growth and metabolism in detail, we employed short hairpin RNAs (shRNAs) synthesized by Jikai Gene to suppress FASN expression. These shRNAs were meticulously designed and successfully cloned into the Lentivirus plasmid pcDNA-EF2-puromycin. Using this system, we were able to selectively silence the FASN gene, further exploring its regulatory mechanisms in cellular functions. To ensure effective delivery and action of shRNAs, Lipofectamine 2000 transfection reagent was chosen for transfection experiments due to its efficient transfection performance, facilitating high-level expression of shRNAs within cells. Following transfection, the effectiveness of the process was assessed through molecular biology techniques such as Western blotting and RT-qPCR. In terms of culture conditions, we opted for Dulbecco's modified Eagle medium supplemented with 10 % fetal bovine serum, maintained at 37 °C and 5 % CO_2_. These culture conditions provided an optimal growth environment for cells, promoting the maintenance of their normal physiological state and ensuring the repeatability and stability of experimental results.

### 2.8 RNA extraction and QPCR

In the pivotal steps of this study, the successful application of TRIzol reagent not only facilitated cell lysis but also enabled efficient purification of RNA through sequential addition of chloroform, 2-propanol, and 75 % ethanol. This ensured the acquisition of high-quality, purified RNA samples, laying a reliable foundation for subsequent experiments. The rigorous examination of the purity and concentration of purified RNA samples is a crucial step to ensure the accuracy of experimental data. The utilization of the Nanodrop 2000 spectrophotometer allows researchers to precisely assess the quality of RNA samples, providing essential information for further analysis and experimental design. During the cDNA synthesis phase, the choice to use 2 μg of purified RNA in conjunction with oligo dT primers and the RevertAid First Strand cDNA Synthesis Kit was made to ensure the generated cDNA possesses high quality and representative expression. This not only aids in preserving the integrity of RNA but also enhances the reliability of subsequent PCR amplification. PCR amplification represents a pivotal stage in the experiment, where precise control of temperature and time allows for the selective amplification of specific gene segments. The optimized PCR conditions, such as the initial incubation at 95 °C, denaturation at 94 °C, annealing at 55 °C, and extension at 72 °C, not only increase the yield of PCR products but also ensure the specificity and stability of the amplification. Primers' specific information was listed as follows: FASN forward primer (5‐AAGGACCTGTCTAGGTTTGATGC‐3) and FASN reversed primer (5‐TGGCTTCATAGGTGACTTCCA‐3); GAPDH (GAPDH, an endogenous control) forward primer (5‐CTGGGCTACACTGAGCACC‐3) and GAPDH reversed primer (5‐AAGTGGTCGTTGAGGGCAATG‐3).

### 2.9 Cell counting Kit-8 (CCK-8)

In this experiment, we utilized a concentration of 2 × 10^4 cells per milliliter, allocating them into a 96-well plate with sufficient experimental space, allowing for concurrent experiments or testing of cells' responses under different conditions. The 96-well plate provided a versatile platform for conducting multiple experiments simultaneously. The Cell Counting Kit-8 (CCK-8) employed in this study is manufactured by Dojindo, based in Kumamoto, Japan. This solution is widely used for assessing cell viability, relying on the measurement of cellular metabolic activity. By adding 10 μL of CCK-8 solution to each well at predefined time points, we were able to monitor changes in cell metabolic activity throughout the experimental process. After a 2-h incubation period, we employed a microplate reader to measure absorbance at 450 nm. This specific wavelength measurement was chosen to accurately assess the reaction products of CCK-8, reflecting the cellular viability. Recording absorbance values provided us with data, enabling the construction of a cell viability curve. The cell viability curve is a dynamic graphical representation illustrating the survival status of cells at different time points. Through this approach, we could observe variations in cell viability over time, providing crucial insights into how cells respond under diverse conditions.

### 2.10 Colony formation

In the experiments conducted using six-well plates, an initial inoculation of 500 cells was performed in each well. Following cell adhesion, various treatments were applied. After a period of 10–14 days, the counting of clones was assessed. The cleaning of clones was carried out using phosphate-buffered saline (PBS), followed by fixation with formaldehyde for 30 min. Crystalline purple was used for staining the six-well plates, and the plates were then set aside for an additional 20 min. At the outset of the experiments utilizing six-well plates, 500 cells were initially seeded into each well, marking the commencement of the study. After allowing the cells to adhere to the plate surface, different treatments were introduced to observe their effects. After a duration of 10–14 days, an evaluation of clone proliferation was conducted. To ensure precise analysis, a meticulous protocol was employed for clone processing. Thorough washing with PBS was carried out to remove any residual substances. Subsequently, fixation of clones was achieved by treating them with formaldehyde for a duration of 30 min. In the subsequent steps, staining of the six-well plates was performed using crystalline purple, providing a visual marker for the assessment of cell clones. This staining process was executed with precision, and the plates were left aside for an additional 20 min to ensure optimal color development.

### 2.11 Cell apoptosis evaluation

Then, we examined the occurrence of cell apoptosis in two specific cell lines, namely RT4 and SW780. To initiate the experimental process, these cultured cells underwent treatment with 20 μmol/l proteinase K from Sigma, followed by fixation using 4 % paraformaldehyde. The evaluation of cell apoptosis was conducted through the terminal transferase UTP nick-end labeling (TUNEL) assay, using a kit provided by Roche and following the recommended protocol from the kit manufacturer. Notably, in the TUNEL assay, apoptotic cells were marked with red fluorescence, which serves as a distinctive feature of this experiment. Subsequently, visualization of these fluorescently labeled cells was performed using an inverted fluorescence microscope. Following visualization, detailed analysis of the obtained images was carried out using the Image J software developed by the National Institutes of Health (NIH). This comprehensive approach allows for a detailed exploration of the cellular response to experimental conditions in RT4 and SW780 cells, providing valuable insights into the apoptotic status of the cells.

### 2.12 Wound healing assay

In this experimental procedure, RT4 and SW780 cells in the logarithmic phase were meticulously prepared by seeding them in six‐well plates, followed by incubation at 37 °C until the cell monolayer achieved an approximate 90 % confluence. The subsequent step involved the initiation of a wound healing assay, wherein uniform and straight scratches were carefully introduced into the cell monolayers using pipette tips. To ensure the removal of any cellular debris, the scratched cell monolayers underwent a thorough wash with phosphate‐buffered saline (PBS). Subsequently, a crucial post-treatment phase unfolded, involving the incubation of cells with 2 mL complete culture medium for an additional 24 h. The dynamic process of wound healing was diligently observed and documented through the acquisition of images at both the initiation of the assay (0 h) and after the 24-h incubation period. To quantify and analyze the rate of wound healing, ImageJ software, a powerful tool developed by the National Institutes of Health (NIH), was employed. This comprehensive approach not only facilitated the visualization of the wound healing process but also enabled a quantitative assessment, providing valuable insights into the migratory and regenerative capabilities of RT4 and SW780 cells under the experimental conditions.

### 2.13 Cell invasion assays

In preparation for invasion assays, Matrigel sourced from BD Biosciences, CA, underwent a meticulous dilution process to achieve a concentration of 1 mg/mL, utilizing DMEM without FBS medium. This appropriately diluted Matrigel was promptly applied to the upper chamber of the *trans*-well system. Following this application, a period of hydration lasting 3–4 h ensued. Subsequently, 1 × 10^5 cells in 200 μl of DMEM without FBS medium were carefully introduced into the upper chamber. Simultaneously, the lower chamber received an addition of 800 μl DMEM supplemented with 10 % FBS per well, creating a conducive environment for cell migration. The assembled *trans*-well system was then subjected to a 24-h incubation period at 37 °C and 5 % CO_2_. Post-incubation, cells that had traversed to the bottom surface of the membrane were meticulously fixed with 100 % methanol for a duration of 30 min. Subsequent to fixation, a staining step ensued, involving the application of 0.1 % crystal violet for 10 min. Following staining, a thorough wash with water was performed three times to remove excess stain. The final step involved the counting and imaging of invading cells, a process carried out in at least 10 random fields under a light microscope. It is noteworthy that this comprehensive invasion assay protocol was executed three times independently, ensuring the reliability and consistency of the obtained results.

## 3 Western blotting

In this experiment, transfected BC cells (BC cells) were lysed, and total protein was extracted using RIPA buffer, followed by quantification using a BCA kit. Subsequently, each protein sample (30 μg) underwent denaturation in sample buffer at 100 °C for 5 min and was subjected to 10 % sodium dodecyl sulfate-polyacrylamide gel electrophoresis for 50 min. The gel was then transferred onto a nitrocellulose membrane at a constant current of 200 mA for 60 min. After blocking the nitrocellulose membrane with 5 % skim milk at room temperature for 2 h, it was incubated overnight at 4 °C with a properly diluted primary protein antibody. The following day, the membrane was washed three times with phosphate-buffered saline with Tween 20 (PBST) solution, incubated with a secondary antibody at room temperature for 45 min, and washed again with PBST. Finally, the membrane was developed using an ECL solution to capture the target band. This procedure allows researchers to analyze protein expression levels and detect the presence of specific proteins in the given cell samples.

## 4 Statistical analysis

Graphpad Prism 8.0 and R version 3.5.2 were employed as key tools in this study for the generation of charts and comprehensive data analysis. The research methodology involved a meticulous approach, ensuring that results were derived from a minimum of three independent experiments. To ensure clarity and accuracy, experimental data were concisely presented as means with their respective standard deviations (x ± s). Statistical analysis was utilized to scrutinize the nuances within the data. The widely adopted Student's t-test was used for comparisons between two groups. In scenarios involving more than two groups, a choice was made between Student's t-test or ANOVA, depending on the context, with SPSS serving as the analytical platform. Through Kaplan-Meier analysis, we explored the potential association between FASN expression and survival outcomes in BC patients. An important consideration throughout the entire study was the adoption of a significance threshold of P < 0.05, designating findings below this predetermined level as statistically significant.

## 5 Results

### 5.1 Identification of the DE-ARGs in BC specimens and functional enrichment analysis

**Fig. 1:**
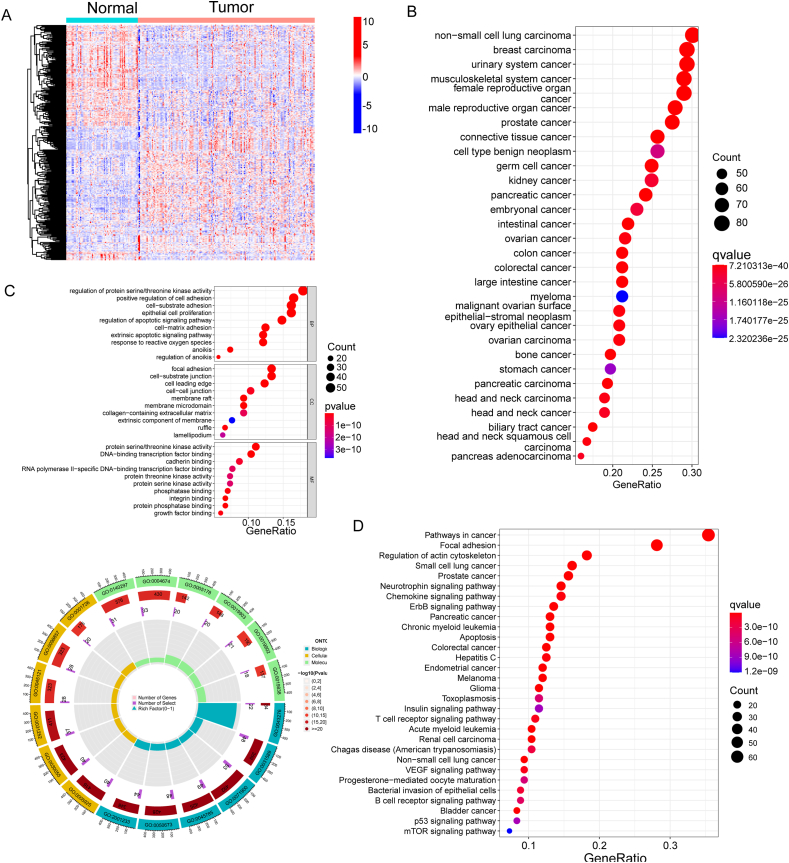
Identification of the DE-ARGs in BC specimens and functional enrichment analysis. (A) The analysis of GSE13507 datasets (including 67 normal samples and 165 tumor samples) using Student's t-test revealed 300 DE-ARGs in BC samples, including 149 downregulated genes and 151 upregulated genes. (B) Disease Ontology (DO) Analysis of DE-ARGs. The distribution of 300 DE-ARGs across various diseases, as shown in the DO analysis. (C) Gene Ontology (GO) Analysis of DE-ARGs. Functional enrichment analysis depicting the biological processes associated with the 300 DE-ARGs. (D) Kyoto Encyclopedia of Genes and Genomes (KEGG) Analysis of DE-ARGs. Pathway enrichment analysis indicating the main pathways associated with the 300 DE-ARGs.

To systematically assess the genetic landscape associated with BC progression, we performed an in-depth analysis of the GSE13507 datasets employing the Student's t-test. Our investigation unveiled 300 DE-ARGs in BC samples, delineating 149 genes experiencing downregulation and 151 genes undergoing upregulation, as depicted in Fig. 1A. Then, we performed DO analysis to explore the possible association between 300 DE-ARGs and various diseases. As shown in Fig. 1B, we found that 300 DE-ARGs were mainly enriched in non-small cell lung carcinoma, breast carcinoma, urinary system cancer, musculoskeletal system cancer, female reproductive organ cancer and male reproductive organ cancer. The results of GO analysis revealed that 300 DE-ARGs were mainly related to regulation of protein serine/threonine kinase activity, positive regulation of cell adhesion, cell-substrate adhesion, epithelial cell proliferation, focal adhesion, cell−substrate junction, cell leading edge, cell−cell junction, protein serine/threonine kinase activity, DNA-binding transcription factor binding, cadherin binding (Fig. 1C). The results of KEGG analysis indicated that 300 DE-ARGs were mainly related to Pathways in cancer, Focal adhesion, Regulation of actin cytoskeleton, Small cell lung cancer and Prostate cancer (Fig. 1D). Our findings provide a comprehensive insight into the altered genetic landscape during BC progression, pinpointing potential molecular markers and pathways that could be instrumental in advancing our understanding and potential therapeutic strategies for BC.

### 5.2 Identification and validation of diagnostic feature biomarkers

**Fig. 2:**
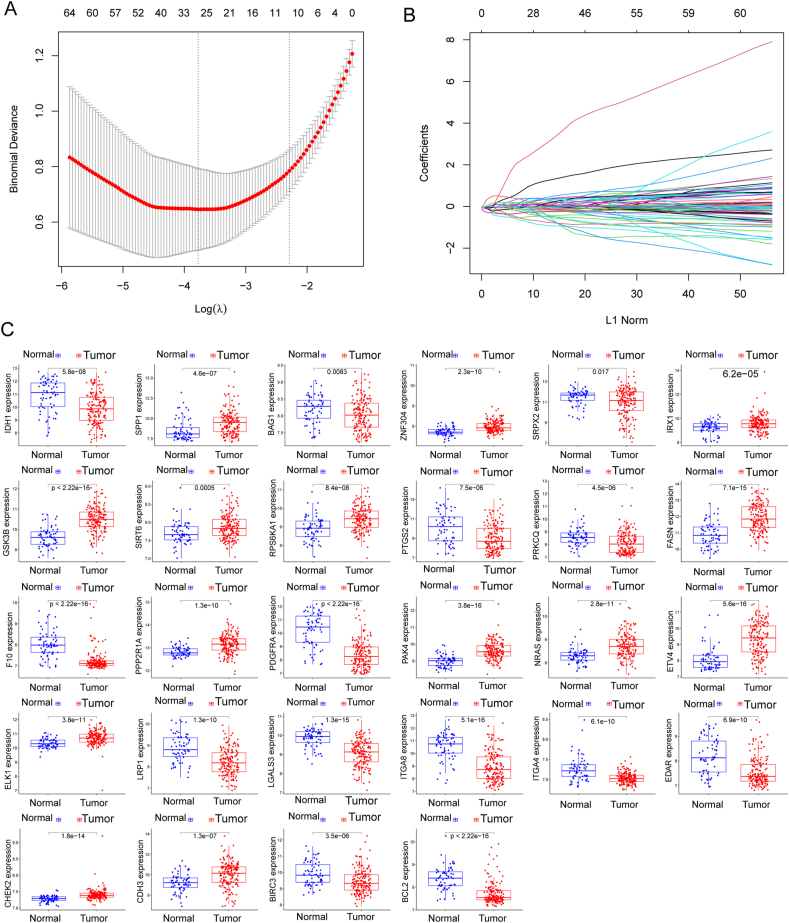
Identification of BC-Related biomarkers Using LASSO Algorithm. (A and B)Application of the LASSO logistic regression algorithm on the GSE13507 dataset, with penalty parameter tuning through 10-fold cross-validation, resulted in the selection of 28 BC-related features. (C) Expression Patterns of 28 Novel Diagnostic Genes. Visualization of the expression patterns of the 28 identified BC-related features, providing insights into their potential diagnostic significance.

**Fig. 3:**
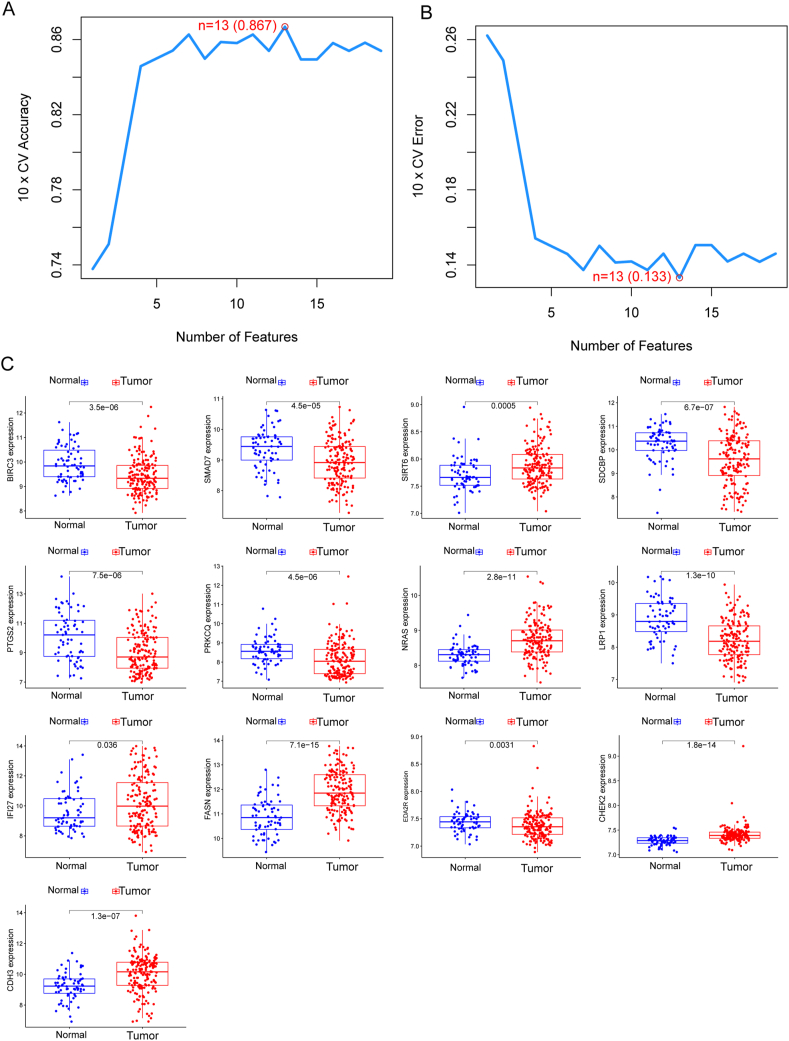
Selection of Optimal Feature Genes Using SVM-RFE Algorithm. (A and B) The SVM-RFE algorithm was applied to filter DE-ARGs, identifying an optimal combination of 13 feature genes. (C)Expression Patterns of 13 Optimal Feature Genes. Visual representation of the expression patterns of the 13 optimal feature genes, offering insights into their potential diagnostic utility.

**Fig. 4:**
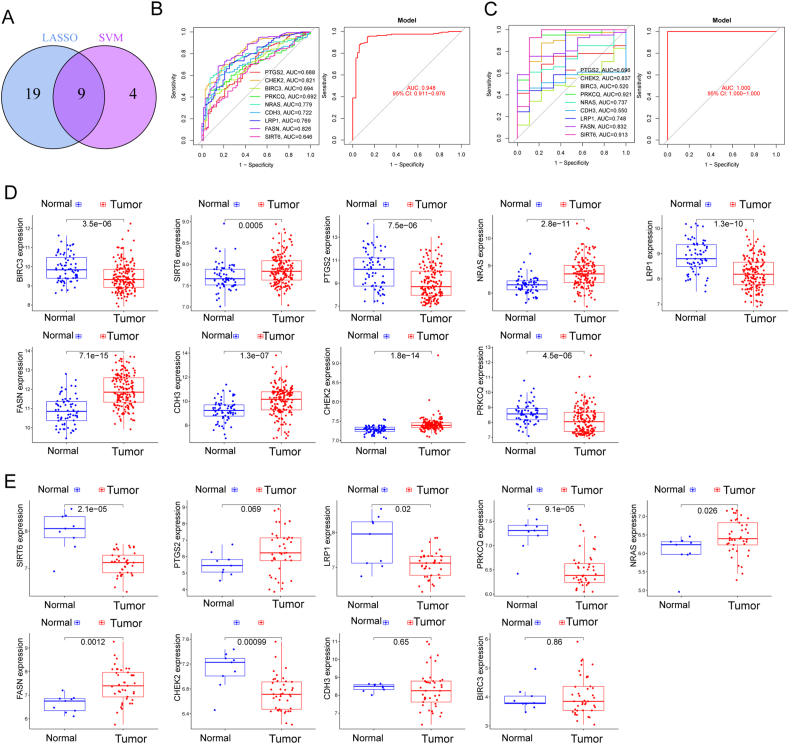
Identification of 9 Marker Genes and the development of a novel diagnostic model. (A) Intersection of marker genes obtained from LASSO and SVM-RFE models, resulting in the selection of 9 marker genes (PTGS2, CHEK2, BIRC3, PRKCQ, NRAS, CDH3, LRP1, FASN, and SIRT6) for further analysis. (B) Evaluation of Logistic Regression Model. Construction of a logistic regression model based on the 9 identified marker genes, demonstrating its effectiveness in differentiating between normal and BC samples with an AUC of 0.948 in ROC curves. (C) Validation in GSE3167 Datasets. Assessment of the diagnostic capability of the logistic regression model in GSE3167 datasets, confirming its robustness. (D and E)Expression patterns of 9 marker genes in BC Specimens. Visualization of the expression patterns of the 9 marker genes in BC specimens from both GSE13507 and GSE3167 datasets.

In our quest to discern variations between BC and normal specimens, we sought to evaluate the diagnostic potential of Differentially Expressed Annotated Genes (DE-ARGs). Employing two distinct machine learning algorithms, namely LASSO and SVM-RFE, on the GSE13507 dataset, we aimed to identify significant DE-ARGs capable of distinguishing BC from normal specimens. The LASSO logistic regression algorithm, incorporating penalty parameter tuning through 10-fold cross-validation, revealed 28 BC-related features (depicted in Fig. 2A and B). The expression patterns of these 28 novel diagnostic genes are illustrated in Fig. 2C. Subsequently, the SVM-RFE algorithm was applied to filter DE-ARGs and identify an optimal combination of feature genes, resulting in the selection of 13 genes (Fig. 3A and B), whose expression patterns are visualized in Fig. 3C. By intersecting the marker genes obtained from the LASSO and SVM-RFE models, we identified 9 marker genes (PTGS2, CHEK2, BIRC3, PRKCQ, NRAS, CDH3, LRP1, FASN, and SIRT6) for further analysis (depicted in Fig. 4A). Utilizing the R package glm, we constructed a logistic regression model based on these 9 marker genes. ROC curves demonstrated that the logistic regression model effectively differentiated between normal and BC samples, achieving an AUC of 0.948 (Fig. 4B). Additionally, we assessed the diagnostic capability of individual genes through ROC curves, all of which exhibited AUC values exceeding 0.65. The robustness of the new model was further validated in the GSE3167 datasets (Fig. 4C). Expression patterns of the 9 marker genes in BC specimens from both GSE13507 (Fig. 4D) and GSE3167 (Fig. 4E) datasets were also elucidated. In summary, our findings suggest that the logistic regression model, incorporating the 9 identified marker genes, provides superior accuracy and specificity in distinguishing BC samples from normal samples compared to individual marker genes.

### 5.3 The expression and prognostic value of 9 marker genes in BC based on TCGA datasets

**Fig. 5:**
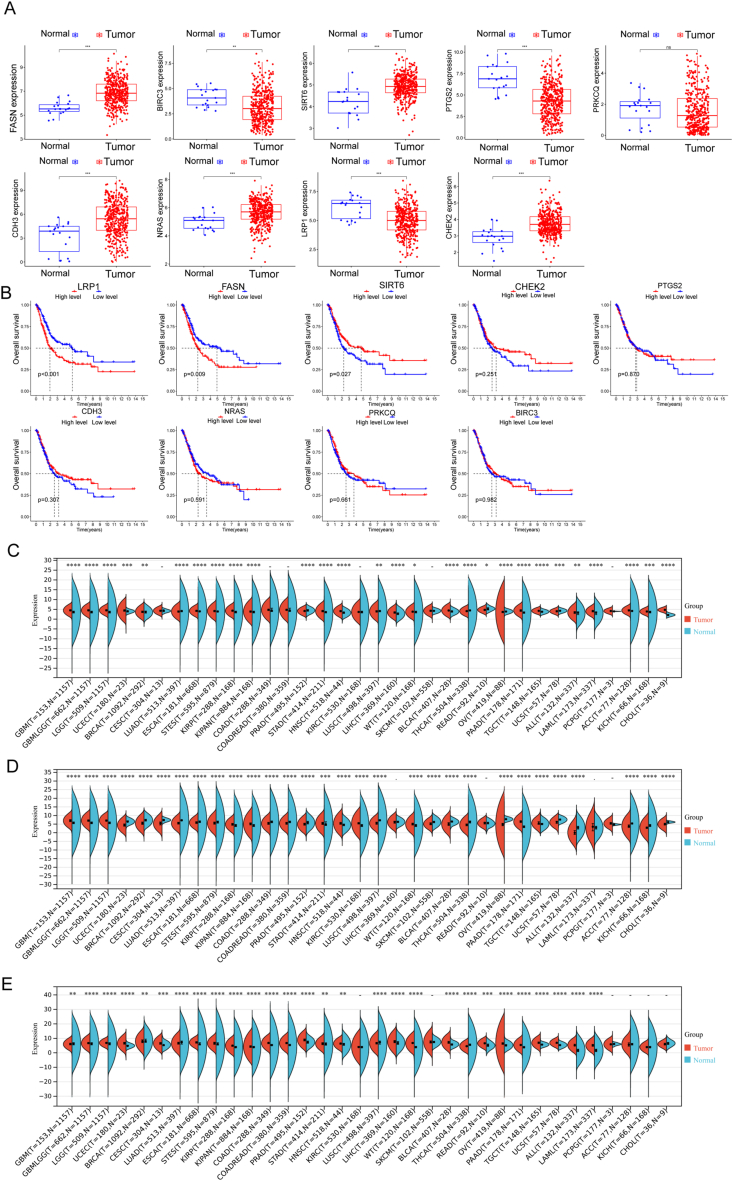
Identification of the critical survival-related marker genes in BC based on TCGA datasets. (A) Expression Profiles of 9 Marker Genes in BC Specimens based on TCGA datasets (including 19 normal samples and 412 tumor samples). (B) Prognostic Value of LRP1, FASN, and SIRT6 in BC Patients. Survival analysis demonstrated a significant association between LRP1, FASN, and SIRT6 expression levels and the clinical outcomes of BC patients. (C–E) Pan-Cancer Analysis of SIRT6, LRP1, and FASN. Examination of dysregulated expression patterns of SIRT6, LRP1, and FASN across various tumor types. *p < 0.05, **p < 0.01, ***p < 0.001.

Subsequently, we scrutinized the expression profiles of the 9 identified marker genes in BC using TCGA datasets. The findings revealed aberrant levels in eight marker genes within BC specimens, namely PTGS2, BIRC3, PRKCQ, NRAS, CDH3, LRP1, FASN, and SIRT6 (depicted in Fig. 5A). Subsequent survival analysis unveiled that LRP1, FASN, and SIRT6 were significantly associated with the clinical outcomes of BC patients (Fig. 5B). Additionally, a pan-cancer analysis demonstrated dysregulated expression patterns of SIRT6 (Fig. 5C), LRP1 (Fig. 5D), and FASN (Fig. 5E) across multiple tumor types. The dysregulated expression observed in various cancers positions SIRT6, LRP1, and FASN as potential candidates for cancer biomarkers. This implies a potential association between the expression levels of these genes and cancer development, clinical progression, or treatment response. The consistent dysregulation observed across diverse cancer types suggests shared biological features or pathways, offering crucial insights for understanding common mechanisms in cancer development and facilitating the design of cross-cancer treatment strategies.

### 5.4 The prognostic value of FASN expression in BC patients

**Fig. 6:**
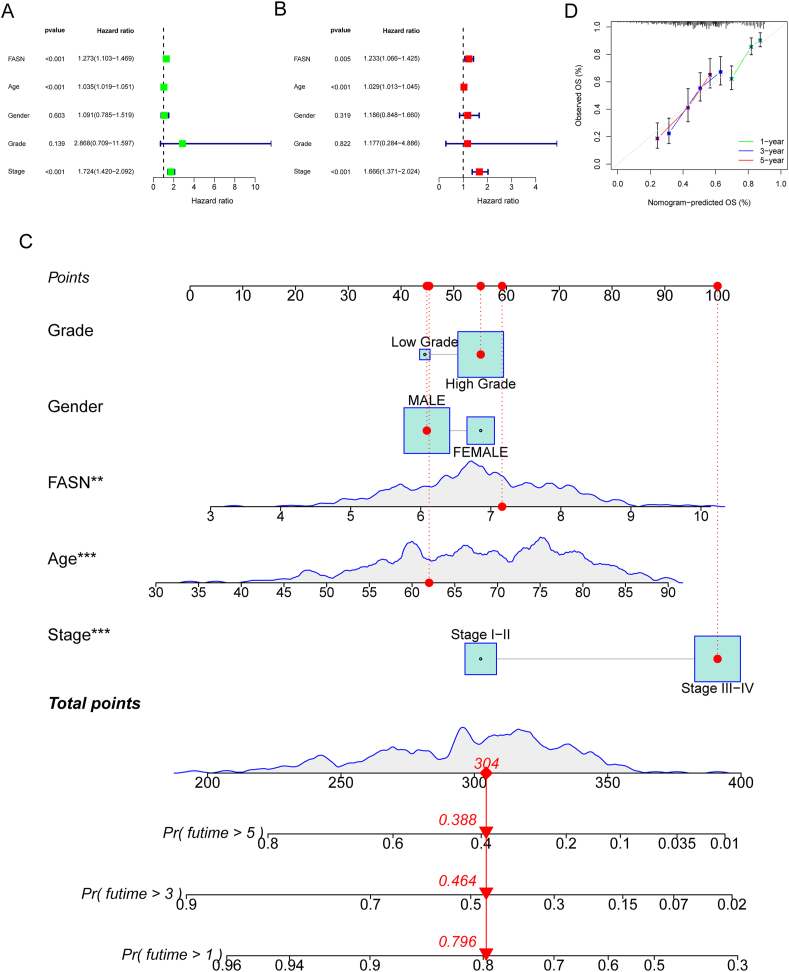
The prognostic values of FASN expressions in BC patients. (A) Univariate Analysis of Prognostic Factors in BC Patients. Univariate analysis using Cox's proportional hazard model illustrating the associations between overall survival in BC patients and key variables. (B) Multivariate Cox Regression Analysis of FASN Expression. Multivariate Cox regression analyses confirming the independent prognostic value of FASN expression for overall survival in BC patients. (C and D) Nomogram model for survival prediction in BC Patients. Development of a nomogram model integrating age, clinical stage, and FASN expression as factors to provide a comprehensive and individualized assessment of overall survival in BC patients. *p < 0.05, **p < 0.01, ***p < 0.001.

Given the limited reports on the role of FASN expression in BC, our attention was directed towards a more in-depth exploration of FASN. To ascertain the potential of FASN as a novel biomarker for BC patients, we conducted comprehensive univariate and multivariate analyses using Cox's proportional hazard model. The univariate analysis revealed significant associations between overall survival of BC patients and variables such as age, clinical stage, and FASN expression (depicted in Fig. 6A). Further strengthening this association, the multivariate Cox regression analyses affirmed that FASN expression stands as an independent prognostic indicator for overall survival in BC patients (Fig. 6B). Considering the diverse clinical factors influencing BC patients, such as age, clinical stage, and FASN expression, and recognizing the need for a practical tool, we developed a nomogram model. This model aims to assist clinicians by providing a more comprehensive and individualized assessment of the survival prospects for patients with BC (illustrated in Fig. 6C and D). The nomogram integrates multiple factors to create a visual and user-friendly tool that can aid in predicting the overall survival of BC patients, thereby contributing to more informed clinical decision-making.

### 5.5 The association between FASN expression and clinical factors of BC patients

**Fig. 7:**
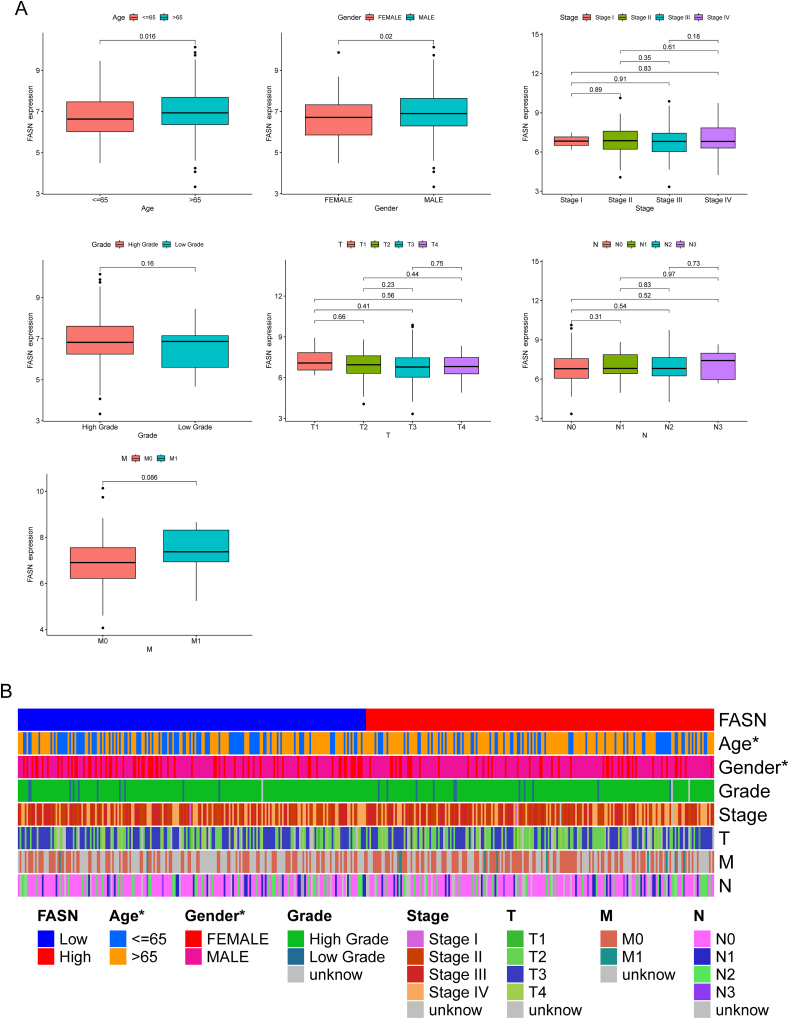
Association between FASN expression and clinical parameters in BC. (A) Exploration of the correlations between FASN expression levels and key clinical factors, including age, gender, stage and grade. (B) Heatmap of Clinical Variables and FASN Expression. Construction of a heatmap visualizing the distribution of clinical variables among individuals with high or low FASN expression. *p < 0.05, **p < 0.01, ***p < 0.001.

Considering the prognostic significance of FASN in BC patients, our objective was to unravel the specific correlations between FASN expression levels and key clinical parameters in BC. We specifically focused on variables such as age, grade, clinical stage, T stage, M stage, and N stage. Notably, we observed that low expression of FASN was notably associated with age and gender, although no significant associations were found with other clinical factors (depicted in Fig. 7A). To provide a visual representation of the variations in the distribution of clinical variables among individuals with high or low FASN expression, we constructed a heatmap illustrating these differences (Fig. 7B). This heatmap serves as a comprehensive tool to highlight the nuanced relationships between FASN expression levels and various clinical parameters, shedding light on potential associations that may contribute to a more thorough understanding of the role of FASN in BC.

### 5.6 The biological functions of FASN in BC

**Fig. 8:**
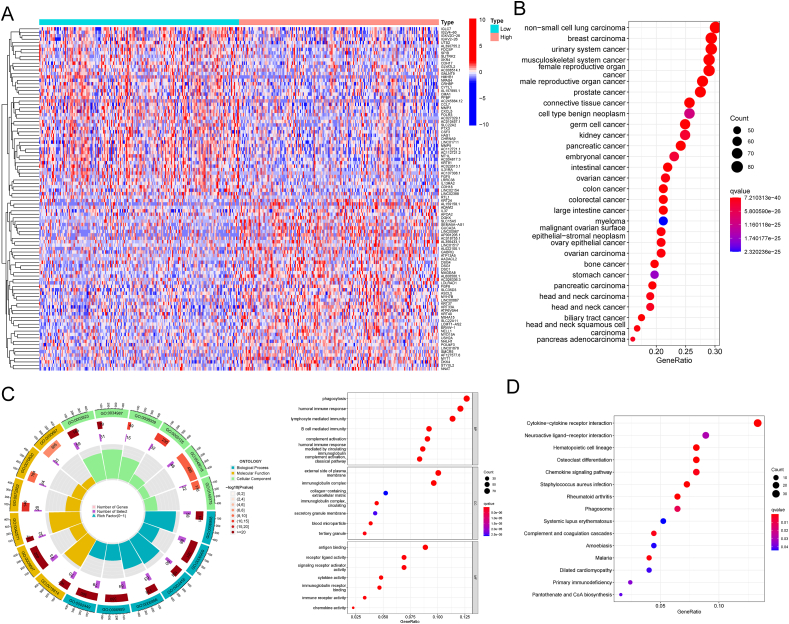
The Biological Functions of FASN in BC using Functional enrichment analysis. (A) Identification of DEGs Associated with FASN Levels. (B) DO Analysis of 1421 DEGs. (C) GO Analysis of 1421 DEGs. (D) KEGG Analysis of 1421 DEGs.

Subsequently, we identified a diverse set of DEGs from individuals characterized by either elevated or diminished FASN levels. A total of 1421 DEGs were discerned through our screening process (depicted in Fig. 8A). Then, we performed DO analysis and found that 1421 DEGs were mainly enriched in non-small cell lung carcinoma, breast carcinoma, urinary system cancer, musculoskeletal system cancer, female reproductive organ cancer and male reproductive organ cancer (Fig. 8B). The results of GO analysis revealed that 1421 DEGs were mainly related to phagocytosis, humoral immune response, lymphocyte mediated immunity, external side of plasma membrane, immunoglobulin complex, collagen-containing extracellular matrix, antigen binding, receptor ligand activity and signaling receptor activator activity (Fig. 8C). The results of KEGG analysis revealed that 1421 DEGs were mainly related to Cytokine-cytokine receptor interaction, Neuroactive ligand-receptor interaction, Hematopoietic cell lineage and Osteoclast differentiation (Fig. 8D).

### 5.7 Knockdown of FASN suppressed BC progression via Wnt/β‐catenin pathway

**Fig. 9:**
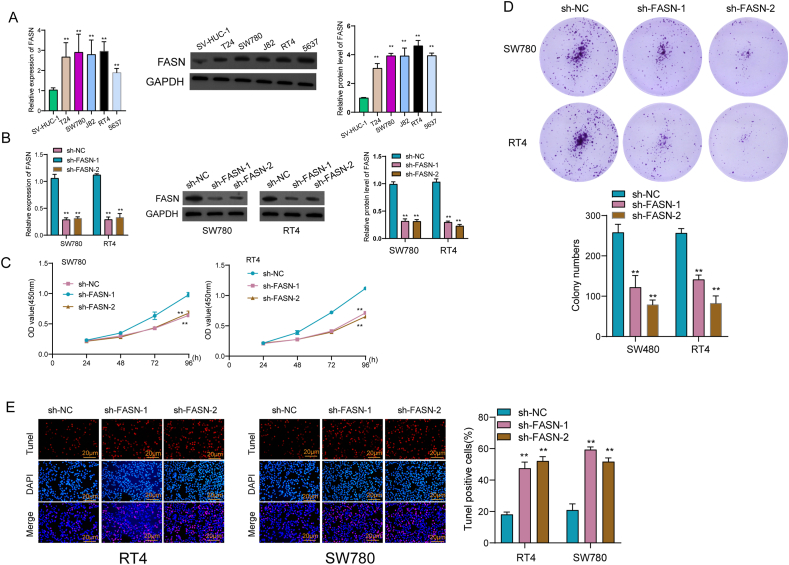
Knockdown of FASN distinctly suppressed the proliferation of BC cells. (A) Elevated Expression of FASN in BC Cell Lines by RT-PCR and Western blot. The original Western blot was shown in Supplementary S1A. (B) Efficient Knockdown of FASN in RT4 and SW780 Cells. Implementation of shRNA plasmids to achieve a robust knockdown of FASN expression in RT4 and SW780 cells. The original Western blot was shown in Supplementary S1B. (C) CCK-8 assays. (D)Clonogenic assays. (E) TUNEL assays. Scale bars: 20μm. *p < 0.05, **p < 0.01, ***p < 0.001.

**Fig. 10:**
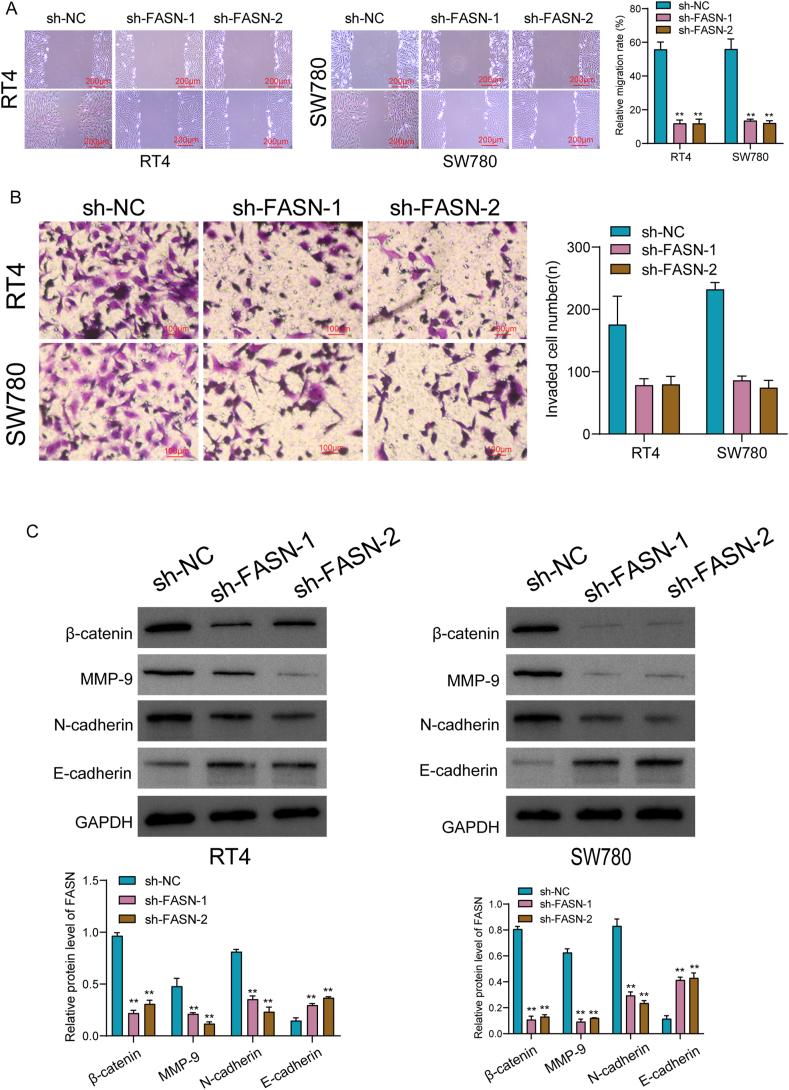
Knockdown of FASN distinctly suppressed the metastasis of BC cells via Wnt/β‐catenin/EMT Pathway. (A) Wound healing experiment highlighting a substantial reduction in the migratory capacity of BC cells following FASN knockdown. Scale bars: 200 μm. (B) Diminished Invasive Ability After FASN Knockdown by transwell assays. Scale bars: 100 μm. (C) Modulation of Wnt/β‐catenin/EMT Pathway proteins by FASN silencing using Western blot. The original Western blot was shown in Supplementary S1C. *p < 0.05, **p < 0.01, ***p < 0.001.

The exploration of FASN's potential role in BC cells commenced with an examination of its expression across various BC cell lines. Remarkably, both FASN mRNA and protein levels exhibited distinct elevation in five different BC cell lines, as depicted in Fig. 9A and Supplementary S1A. Given that the expression of FASN exhibited a higher level in RT4 and SW780 cells, we chose them as further experiments. To investigate the functional implications, we employed shRNA plasmids to effectively knock down FASN expression in RT4 and SW780 cells, achieving an efficiency exceeding 60 %, as demonstrated in Fig. 9B and Supplementary S1B. Subsequent assessments through CCK-8 assays and clonogenic assays unequivocally revealed that the knockdown of FASN significantly impeded the proliferation of RT4 and SW780 cells (Fig. 9C and D). Furthermore, TUNEL assays demonstrated a notable increase in apoptosis upon FASN knockdown in both RT4 and SW780 cells (Fig. 9E). Expanding our inquiry, we explored the impact of FASN inhibition on the proliferation and metastasis of BC cells. Results from the wound healing experiment highlighted a substantial reduction in the migratory capacity of BC cells following FASN knockdown (Fig. 10A). Further insights were gained through transwell assays, which indicated a diminished invasive ability of RT4 and SW780 cells after FASN knockdown (Fig. 10B). Additionally, we scrutinized the expressions of key proteins associated with the Wnt/β‐catenin/EMT pathway, including N‐cadherin, E‐cadherin, MMP‐9, and β‐catenin. Our findings revealed that the silencing of FASN led to a suppression of N‐cadherin, β‐catenin, and MMP‐9 expressions, while concurrently promoting the expression of E‐cadherin (Fig. 10C and Supplementary S1C).

## 6 Discussion

Clinical tumor diagnostic markers for BC include Bladder Tumor Antigen (BTA), urine cytology (UroVysion), Urothelial Cancer-Associated Antigen 1 (UCA1), Cytokeratin-19 Fragment (CYFRA 21-1), and Bladder Cancer MCM6 marker [22,23]. These markers play diverse roles in the diagnosis, staging, and prognostic assessment of BC. While they exhibit variations in specificity and sensitivity, each has its own advantages and limitations [6,24]. For instance, BTA and UroVysion, though non-invasive, demonstrate relatively lower specificity, whereas UCA1 and MCM6 markers show potential advantages in early diagnosis [25,26]. Overall, the comprehensive use of these markers contributes to improved accuracy and reliability in diagnosing BC, providing valuable information for patient treatment. Despite certain challenges, these markers play a crucial role in facilitating early detection and treatment of BC. Enhancing the sensitivity of biomarkers is imperative for advancing the diagnostic capabilities in BC patients.

Anoikis, translated as “programmed cell death induced by loss of extracellular adhesion,” refers to a form of programmed cell death triggered when cells lose normal adhesion to surrounding cells or the extracellular matrix [[27], [28], [29]]. Cells rely on adhesion to neighboring cells or the matrix to maintain their normal function and survival. When cells lose this adhesion, they may undergo Anoikis, preventing unattached cells from entering other sites and forming aberrant growth or metastatic tumors [30,31]. Under normal circumstances, Anoikis serves as a protective mechanism, contributing to the maintenance of tissue structure stability and preventing the uncontrolled growth and spread of abnormal cells [32,33]. However, in tumors, the Anoikis mechanism is often disrupted, enabling cancer cells to grow and spread to other locations within the body. The resistance of tumor cells to Anoikis is a crucial factor in tumor metastasis. During the development of cancer, some tumor cells may overcome the restrictions of Anoikis, escape from the primary tumor site, enter the bloodstream or lymphatic system, and then settle in other tissues or organs, forming distant metastatic tumors [34,35]. This increases the invasiveness and metastatic potential of tumors, making treatment more challenging. Anoikis have been studied in many cancers, such as pancreatic cancer, gastric cancer, colorectal cancer and [[36], [37], [38], [39]]. However, the specific function of Anoikis-related genes in BC was rarely reported. In this study, we firstly identified 300 DE-ARGs in BC specimens through a comprehensive analysis of GSE13507 datasets. These genes exhibited significant associations with various diseases, particularly non-small cell lung carcinoma, breast carcinoma, and urinary system cancer. GO analysis revealed their involvement in key cellular processes, while KEGG analysis highlighted their association with cancer-related pathways. Furthermore, the research successfully developed a diagnostic model based on 9 identified marker genes, demonstrating superior accuracy and specificity in distinguishing BC from normal samples. This model offers potential insights into the genetic landscape of BC and presents a valuable tool for improved diagnostic precision in clinical settings.

Then, we performed survival analysis using nine diagnostic genes based on TCGA datasets and only found that SIRT6, LRP1, and FASN were associated with clinical prognosis of BC patients. Given that the function of FASN in BC was rarely reported. Then, we focused on FASN. Fatty Acid Synthase (FASN) is a multifunctional enzyme involved in de novo fatty acid synthesis [40]. It plays a crucial role in cellular lipid metabolism by catalyzing the synthesis of long-chain fatty acids from acetyl-CoA and malonyl-CoA [41,42]. FASN is a large, homodimeric protein with multiple functional domains, making it a key player in lipid biosynthesis and energy storage. FASN has garnered significant attention in cancer research due to its association with tumorigenesis and cancer progression [43,44]. Several studies have implicated FASN in various cancers, highlighting its role in providing cancer cells with the necessary fatty acids for membrane synthesis, energy storage, and signaling pathways. The upregulation of FASN is commonly observed in cancer cells, contributing to their uncontrolled growth and survival [[45], [46], [47]]. In this study, we confirmed FASN expression as an independent prognostic indicator for overall survival in BC patients. Moreover, we performed functional experiments and confirmed that FASN was highly expressed in BC cells and its knockdown distinctly suppressed the proliferation and metastasis of BC cells. Our findings suggested FASN as a novel tumor promotor in BC. Therefore, therapeutic strategies targeting FASN may help inhibit the development and metastasis of BC, offering a new direction for the treatment of BC.

Wnt/β-catenin is a signaling pathway that plays a crucial role in various cellular processes, including embryonic development, tissue homeostasis, and regulation of gene expression [[48], [49], [50]]. The pathway is initiated by Wnt ligands, which bind to cell surface receptors, leading to the activation of intracellular signaling cascades. In the absence of Wnt signaling, a “destruction complex” is formed, which targets β-catenin for degradation [51]. When Wnt ligands bind to their receptors, the destruction complex is inhibited, allowing β-catenin to accumulate in the cytoplasm. Subsequently, β-catenin translocates into the nucleus, where it interacts with transcription factors of the T-cell factor/lymphoid enhancer factor (TCF/LEF) family [52,53]. This interaction leads to the activation of target genes involved in cell proliferation, survival, and differentiation. Aberrant activation of the Wnt/β-catenin pathway is associated with various diseases, including cancer [54,55]. In cancer, mutations or dysregulation of components in the pathway can result in the constitutive activation of Wnt signaling, promoting tumor growth and metastasis. The Wnt/β-catenin pathway is a key player in cancer stem cell maintenance, epithelial-mesenchymal transition (EMT), and resistance to apoptosis. It has been confirmed that the aberrant activation of the Wnt/β-catenin pathway in BC may promote tumor development and progression through various mechanisms [[56], [57], [58]]. In this study, we performed Western blot and found that the silencing of FASN led to a suppression of N‐cadherin, β‐catenin, and MMP‐9 expressions, while concurrently promoting the expression of E‐cadherin, suggesting that FASN may promote the proliferation and metastasis via modulating Wnt/β-catenin pathway.

This article has a number of limitations. Firstly, the study heavily relies on the analysis of the GSE13507 and GSE3167 datasets. The representativeness of these datasets may be limited, and results might not be universally applicable to all BC cases. Secondly, while the study explores the functional role of FASN in BC cell lines, the findings primarily focus on cell proliferation, apoptosis, migration, and invasion. Further investigations into the underlying molecular mechanisms and in vivo studies are necessary for a comprehensive understanding of FASN's role in BC progression.

## 7 Conclusion

We identified 300 differentially expressed annotated genes (DE-ARGs) closely associated with BC progression. These genes are predominantly enriched across various cancers, participating in the regulation of protein kinase activity, cell adhesion, and other biological processes. Utilizing machine learning algorithms, we identified 28 BC-related features and constructed a logistic regression model based on 9 marker genes, demonstrating superior accuracy in distinguishing BC from normal samples. Validation in TCGA datasets revealed significant correlations between LRP1, FASN, SIRT6, and clinical outcomes in BC patients, suggesting their potential as cancer biomarkers. FASN, identified as an independent prognostic indicator, regulates BC cell proliferation and metastasis through the Wnt/β-catenin pathway. These findings provide crucial insights for a deeper understanding of BC's molecular mechanisms and future therapeutic strategies.

## Ethics approval and consent to participate

Not applicable.

## Consent for publication

Not applicable.

## Data availability statement

The datasets analyzed during the current study are available in The Cancer Genome Atlas database (TCGA, https://tcga-data.nci.nih.gov/tcga/) and the Gene Expression Omnibus database (GEO, https://www.ncbi.nlm.nih.gov/geo/) [GSE13507 and GSE3167 datasets]. The datasets generated during and/or analyzed during the current study are available from the corresponding author upon reasonable request.

## Fundings

This research did not receive specific grants or fundings.

## CRediT authorship contribution statement

**Ruoyu Peng:** Writing – review & editing, Writing – original draft, Visualization, Software, Resources, Project administration, Investigation, Formal analysis, Data curation, Conceptualization. **Xiaohan Ma:** Writing – review & editing, Writing – original draft, Visualization, Software, Resources, Methodology, Investigation, Data curation, Conceptualization. **Zhiyun Jiang:** Writing – review & editing, Writing – original draft, Visualization, Resources, Project administration, Methodology, Investigation, Data curation, Conceptualization. **Yu Duan:** Writing – review & editing, Writing – original draft, Visualization, Resources, Project administration, Methodology, Investigation, Formal analysis, Data curation, Conceptualization. **Shaogang Lv:** Writing – review & editing, Writing – original draft, Visualization, Resources, Project administration, Methodology, Data curation, Conceptualization. **Wei Jing:** Writing – review & editing, Writing – original draft, Visualization, Supervision, Project administration, Methodology, Investigation, Formal analysis, Data curation, Conceptualization.

## Declaration of competing interest

The authors declare that they have no known competing financial interests or personal relationships that could have appeared to influence the work reported in this paper.
