# Supplementary material for: Integrative analysis of Anoikis-related genes reveals that FASN is a novel prognostic biomarker and promotes the malignancy of bladder cancer via Wnt/β-catenin pathway
Source: Heliyon. 2024 Jul 3;10(13):e34029. doi: 10.1016/j.heliyon.2024.e34029 (PMC11283158; doi:10.1016/j.heliyon.2024.e34029)
Supplement: Multimedia component 1 [file mmc1.docx]

Supplementary materials

Supplementary S1: Original images of WB assay.


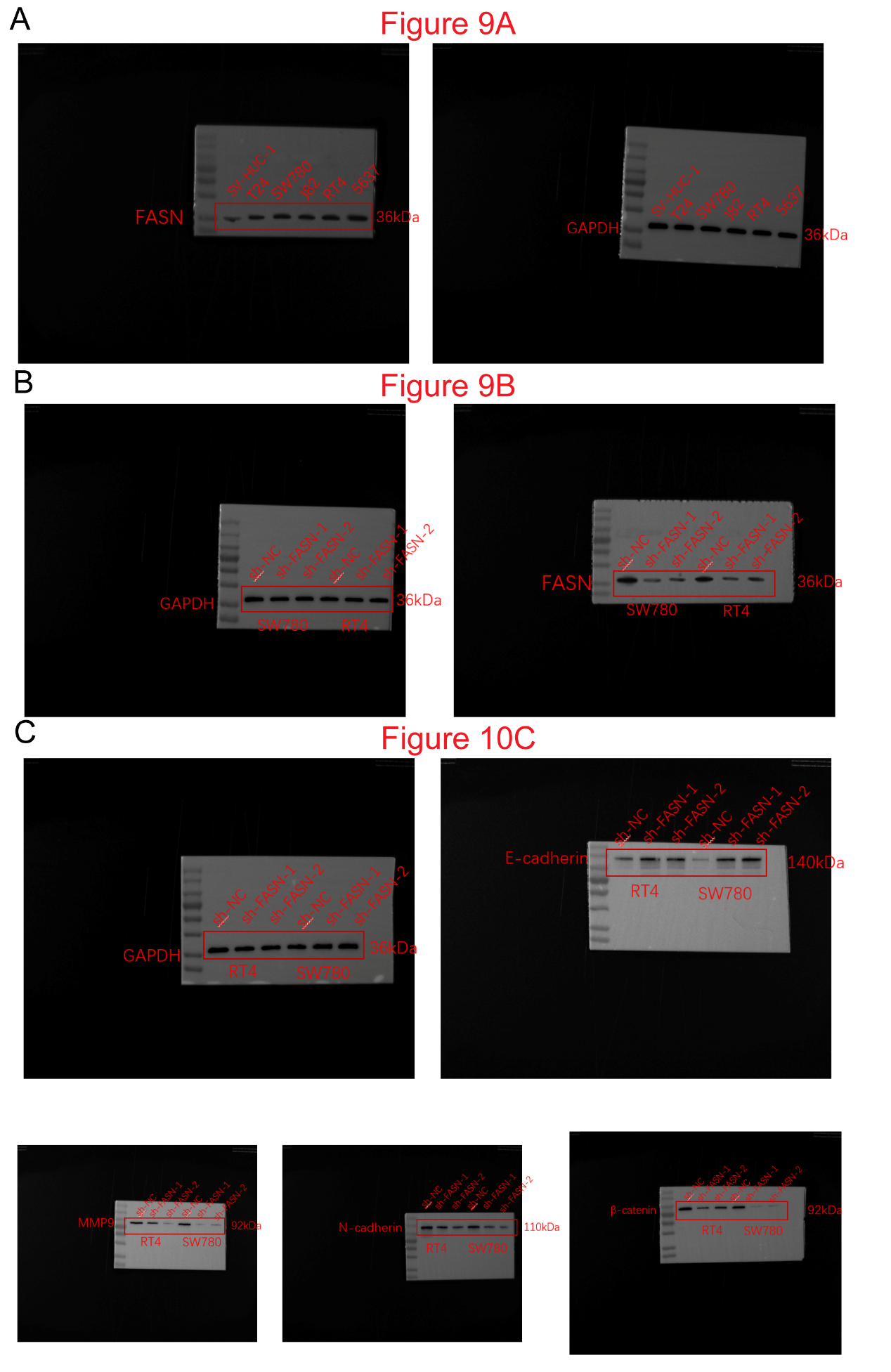


(A) Original images of WB assay of Figure 9A. (B) Original images of WB assay of Figure 9B. (C) Original images of WB assay of Figure 10C.
